# The complete chloroplast genome sequence of *Salix kochiana* Trautv. and its phylogenetic analysis

**DOI:** 10.1080/23802359.2022.2087555

**Published:** 2022-06-23

**Authors:** Jing Wang, Zicheng Yu, Xu Yao, Jie Wan, Zhengxuan Wang, Xiaoping Li

**Affiliations:** a Collaborative Innovation Center of Southern Modern Forestry, Nanjing Forestry University, Nanjing, China; b College of Forestry, Nanjing Forestry University, Nanjing, China; c Jiangsu Key Laboratory for Poplar Germplasm Innovation and Variety Improvement, Nanjing Forestry University, Nanjing, China

**Keywords:** Chloroplast genome, phylogenetic analysis, *Salix kochiana* Trautv., Salicaceae

## Abstract

*Salix kochiana* Trautvetter 1837 is one of the highest value shrubs present in northern China with important economic and ecological benefits. This study revealed the structural characteristics and phylogenetic relationships of chloroplast genes in *S. kochiana* Trautv. The results showed that the length of the complete chloroplast genome was 155,657 bp, which was a typical circular double-stranded structure, including an 84,458 bp large single-copy region (LSC), a 16,221 bp small single-copy region (SSC) and a 27,489 bp pair of inverted repeat regions (IRA and IRB). The chloroplast genome contains 48,757 A bases, 28,017 G bases, 49,843 T bases, and 29,040 C bases, with a GC content of 36.66%. Through bioinformatics annotation, a total of 126 genes were found in the chloroplast genome, including 81 protein-coding genes, 37 tRNA genes, and eight rRNA genes. Phylogenetic analysis showed that *S. kochiana* Trautv. was closely related to *S. triandroides*.

The *Salix* genus is composed of ∼ 350–520 species that are distributed worldwide (Zhou et al. 2021). In China, the *Salix* genus contains 257 species, including 122 varieties and 33 variants, and they are distributed across provinces (Wang et al. 2012). *Salix kochiana* Trautv., an important species of the *Salix* genus, is mainly distributed in the Heilongjiang and Jilin provinces of China and is also found in Mongolia and Russia. *S. kochiana* Trautv. can grow in sand dunes and river wetlands, so it can serve as an important tree species for windbreaks, sand fixation, and soil conservation in northern China. With its fast growth, luxuriant foliage, and slender and soft branches, *S. kochiana* Trautv. can be made into a variety of woven goods. Its twigs and leaves can also be used as animal feed. Therefore, *S. kochiana* Trautv. has high economic value and provides high ecological benefits. However, the available genetic and genomic resources for *S. kochiana* Trautv. are very limited. In addition, due to the highly efficient crossing rate among *Salix* species, the classification of the *Salix* genus is still unclear (Chen et al. 2010). Here, we revealed the structural characteristics and phylogenetic relationships of chloroplast genes in *S. kochiana* Trautv. These results are of great significance for the taxonomic study of *Salix* and even Salicaceae and can also be used to evaluate the breeding availability of *Salix* at the genomic level (Chen and Liu 2008; Ren and Li 2021).

The test materials were collected from Mao’er Mountain of Heilongjiang Province, China (44°29′29″N, 127°17′41″E). The specimens were stored in Room 60708 Biotechnology Building (University sample room), Nanjing Forestry University, China (Xiaoping Li, xpli@njfu.edu.cn, voucher number: MRSSQL2017_1451). The modified CTAB method (cetyl trimethylammonium bromide) (Doyle and Doyle 1987) was used to extract whole-genome DNA from the leaves of *S. kochiana* Trautv., and DNA concentration and purity were detected by 1% agarose gel electrophoresis and a NanoDrop spectrophotometer (Niu et al. 2020). Qualified DNA fragment libraries were constructed with an average insert size of 300 bp using the Illumina Nextera kit and sequenced by the Illumina NovaSeq 6000 platform (Illumina, San Diego, CA). A total of 3.5 G raw data were obtained and filtered to eliminate low-quality reads by Fastp (Chen et al. 2018). Finally, approximately 3.2 G of high-quality clean reads were harvested and used to assemble the chloroplast genome with NOVOPlasty (version 4.1) (https://github.com/ndierckx/NOVOPlasty/) (Dierckxsens et al. 2017). The chloroplast genes of *S. kochiana* Trautv. were aligned and annotated with the chloroplast genome of the reference species *S. triandroides* (MW929215.1) by PGA (Plastid Genome Annotator) (Qu et al. 2019). After manual correction by Geneious v11.0.3 (Kearse et al. 2012), the resulting annotation of the complete chloroplast DNA was obtained and submitted to NCBI online by BankIt (GenBank accession number: OL339478.1).

The annotated results showed that the complete chloroplast genome length was 155,657 bp, which was a typical circular double-stranded structure, including an 84,458 bp large single-copy region (LSC), a 16,221 bp small single-copy region (SSC), and a 27,489 bp pair of inverted repeat regions (IRA and IRB). The chloroplast genome contains 48,757 A bases, 28,017 G bases, 49,843 T bases, and 29,040 C bases, with a GC content of 36.66%. A total of 126 genes were identified, including 81 protein-coding genes, 37 tRNA genes, and eight rRNA genes. Among them, 7 protein-coding genes, 7 tRNA genes and 4 rRNA genes were repeated in the inverted repeat region.

To reveal the relationship between *S. kochiana* Trautv. and other species in the *Salix* genus, we downloaded the chloroplast genome sequences of 19 published *Salix* species from the NCBI database and performed phylogenetic analysis. Comparison of the S. *kochiana* Trautv. plastome to previously published data shows a high level of gene synteny with some publicly available *Salix* sequences (Zhou et al. 2021). *Arabidopsis thaliana* (NC_000932.1) was used as an outgroup. MAFFT v7 software (https://mafft.cbrc.jp/alignment/software/) (Katoh and Standley 2013) was used to perform multiple weight ratio pairs for the common sequences of chloroplast genomes of these 21 species. A phylogenetic tree was constructed based on the maximum likelihood (ML) method with the general time reversible model and 1000 bootstrap replicates using MEGA11 (Koichiro et al. 2021). The results showed that *S. kochiana* Trautv. was most closely related to *S. triandroides* (Figure 1). This finding was similar to those of a previous *Salix* whole-plastome phylogenetic study (Wang and Li 2021; Chen et al. 2019). These results explain the systematic origin and molecular phylogenetic location of *S. kochiana* Trautv., which can be used for molecular identification and resource exploitation. Overall, this study lays a foundation for *Salix* phylogenetics in the future.

**Figure 1.**
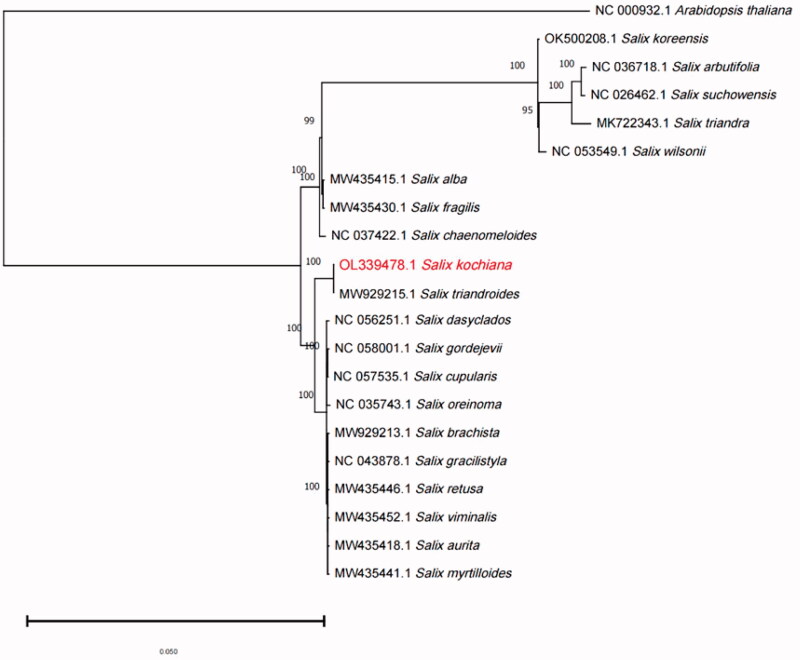
Maximum likelihood phylogenetic tree based on 21 chloroplast genome sequences of selected species. The numbers near branches indicate bootstrap support values.

## Data Availability

The complete chloroplast genome sequence data of *S. kochiana* Trautv. supporting the findings of this study are openly available in Genebank of NCBI at http://www.ncbi.nlm.nih.gov/ under the accession number OL339478.1. The associated BioProject, SRA, and BioSample numbers are PRJNA778909, SRR16924947, and SAMN23003006 respectively.

## Regulation statement

This study strictly follows the wild plant protection regulations of China, which were firstly promulgated by Decree No. 204 of the state council of China on Sept. 30, 1996 and amended on Oct.7, 2017 under Decision of The State Council on Revising Some Administrative Regulations.
